# Quantitative analyses and modelling to support achievement of the 2020 goals for nine neglected tropical diseases

**DOI:** 10.1186/s13071-015-1235-1

**Published:** 2015-12-09

**Authors:** T. Déirdre Hollingsworth, Emily R. Adams, Roy M. Anderson, Katherine Atkins, Sarah Bartsch, María-Gloria Basáñez, Matthew Behrend, David J. Blok, Lloyd A. C. Chapman, Luc Coffeng, Orin Courtenay, Ron E. Crump, Sake J. de Vlas, Andy Dobson, Louise Dyson, Hajnal Farkas, Alison P. Galvani, Manoj Gambhir, David Gurarie, Michael A. Irvine, Sarah Jervis, Matt J. Keeling, Louise Kelly-Hope, Charles King, Bruce Y. Lee, Epke A. Le Rutte, Thomas M. Lietman, Martial Ndeffo-Mbah, Graham F. Medley, Edwin Michael, Abhishek Pandey, Jennifer K. Peterson, Amy Pinsent, Travis C. Porco, Jan Hendrik Richardus, Lisa Reimer, Kat S. Rock, Brajendra K. Singh, Wilma Stolk, Subramanian Swaminathan, Steve J. Torr, Jeffrey Townsend, James Truscott, Martin Walker, Alexandra Zoueva, NTD Modelling Consortium

**Affiliations:** University of Warwick, Coventry, CV4 7AL UK; Liverpool School of Tropical Medicine, Liverpool, L3 5QA UK; Imperial College London, London, W2 1PG UK; London School of Hygiene and Tropical Medicine, London, WC1E 7HT UK; Johns Hopkins Bloomberg School of Public Health, Baltimore, MD 21205 USA; Bill and Melinda Gates Foundation, Seattle, USA; Erasmus University Medical Center, 3015 CE Rotterdam, Netherlands; Princeton University, New Jersey, NJ 08544 USA; Yale University, New Haven, CT 06520 USA; Monash University, Melbourne, VIC 3800 Australia; Case Western Reserve University, Cleveland, OH 44106 USA; University of California, San Francisco, San Francisco, CA 94143 USA; University of Notre Dame, South Bend, IN 47556 USA; Vector Control Research Centre, Pondicherry, 605006 India; Children’s Investment Fund Foundation, London, W1S 2FT UK

**Keywords:** Modelling, Elimination, Neglected tropical diseases, Transmission, Chagas disease, Visceral leishmaniasis, Kala-azar, Human African trypanosomiasis, Leprosy, Soil-transmitted helminths, Schistosomiasis, Lymphatic filariasis, Onchocerciasis, Trachoma, Mass drug administration, Preventive chemotherapy

## Abstract

Quantitative analysis and mathematical models are useful tools in informing strategies to control or eliminate disease. Currently, there is an urgent need to develop these tools to inform policy to achieve the 2020 goals for neglected tropical diseases (NTDs). In this paper we give an overview of a collection of novel model-based analyses which aim to address key questions on the dynamics of transmission and control of nine NTDs: Chagas disease, visceral leishmaniasis, human African trypanosomiasis, leprosy, soil-transmitted helminths, schistosomiasis, lymphatic filariasis, onchocerciasis and trachoma. Several common themes resonate throughout these analyses, including: the importance of epidemiological setting on the success of interventions; targeting groups who are at highest risk of infection or re-infection; and reaching populations who are not accessing interventions and may act as a reservoir for infection,. The results also highlight the challenge of maintaining elimination ‘as a public health problem’ when true elimination is not reached. The models elucidate the factors that may be contributing most to persistence of disease and discuss the requirements for eventually achieving true elimination, if that is possible. Overall this collection presents new analyses to inform current control initiatives. These papers form a base from which further development of the models and more rigorous validation against a variety of datasets can help to give more detailed advice. At the moment, the models’ predictions are being considered as the world prepares for a final push towards control or elimination of neglected tropical diseases by 2020.

## Background

Neglected tropical diseases (NTDs) continue to create significant levels of suffering and morbidity throughout the tropical world. They affect over a billion people and accompany HIV/AIDS, tuberculosis and malaria as the classic ‘diseases of poverty’ [1]. Considerable evidence suggests that NTDs place major constraints on economic development in most tropical countries [2]. The potential for large-scale improvements in health equity by tackling these diseases has been recognised in recent years by large-scale investment in controlling them. In January 2012, the World Health Organization (WHO), laid out a roadmap for controlling the burden of morbidity of neglected tropical diseases [3]. This included goals for achieving control, local elimination “as a public health problem”, or reduction in disease burden to low levels by 2020. The London Declaration on NTDs, signed in 2012, demonstrated the support of the pharmaceutical industry, governments and non-governmental agencies for the achievement of these goals for ten diseases. Of these, one, Guinea worm, was targeted for eradication. The remaining nine, lymphatic filariasis, leprosy, human African trypanosomiasis, blinding trachoma, schistosomiasis, soil-transmitted helminthiasis, Chagas disease, visceral leishmaniasis, and onchocerciasis (Table 1) were targeted for control or “elimination as a public health problem.”. Elimination as a public health problem is defined differently for each disease, with individual disease goals set in accordance with the epidemiology of each disease. Elimination as a public health problem as defined by WHO does not necessarily require a break in transmission, rather a dramatic cut in disease incidence or prevalence.

**Table 1:** Summary of the nine neglected tropical diseases studied in these papers, where elimination refers to elimination *as a public health problem*. Data sources: WHO

Name	Transmission	Global picture	Interventions	WHO target for 2020
Preventive chemotherapy (PCT) diseases, controlled by mass drug administration (MDA) programmes				
Lymphatic filariasis (elephantiasis)	Worm transmitted by mosquito	Tropical and subtropical countries in Africa, Asia, the Western Pacific, the Caribbean and South America	Annual/biannual MDA (ivermectin, albendazole and DEC), vector control through insecticide-treated bed nets or spraying	Global elimination
Onchocerciasis (river blindness)	Worm transmitted by black fly	Primarily occurs in tropical sub-Saharan Africa (99 % of cases)	MDA (ivermectin) and vector control	Country elimination
Schistosomiasis (bilharzia)	Intestinal worm, water-borne transmission with snail intermediate host	Affect at least 240 million people worldwide. Most commonly found in Africa, as well as Asia and South-America	MDA (praziquantel) to school-agechildren and high-risk adults, along with WASH and possible snail control	Regional and country elimination
Soil-transmitted helminthiasis (roundworm, whipworm, hookworm)	Intestinal worms transmitted via soil contaminated with fecal matter	Over 1 billion people affected, particularly in sub-Saharan Africa, India and Southeast Asian countries	MDA (albendazole, mebendazole) treatment of school-aged children. Treatment of pre-school aged children and women of childbearing age is also recommended.	75 % coverage with (bi)annual PCT
Blinding trachoma	Bacterial infection transmitted by flies, fingers and fomites.	84 million active cases globally.	MDA (azithromycin) and surgery, along with improved hygiene	Global elimination
Intensified disease management (IDM) diseases, controlled by increased diagnosis and management of cases				
Chagas disease	Protozoan transmitted by triatomines (kissing bugs)	8 million infected in the Americas, 10,000 deaths per year.	Spraying with indoor residual insecticides, housing improvements.	Regional elimination
HAT (sleeping sickness), Gambian form	Protozoan transmitted by tsetse fly	<4000 new cases in 2014	Treatment, active/mass screening and vector control with tsetse targets.	Global elimination
Leprosy	Bacterium with unclear mode of transmission: contact or droplet likely	200,000 new diagnoses per year, >80 % from India, Brazil and Indonesia	Early diagnosis and treatment	Global elimination
Visceral leishmaniasis (kala-azar) in the Indian sub-continent	Protozoan transmitted by sand fly	200,000–400,000 cases annually, 80 % in Indian sub-continent.	Indoor residual spraying of insecticides, insecticide-treated bed nets, active case detection, rapid diagnosis and treatment	Regional elimination

In the wake of the London Declaration a need has been identified for epidemiological modelling to aid control policy design and evaluation. Although the epidemiological modelling of NTDs has a long history [4, 5], it has been limited by both a lack of interest from funders and limited epidemiological data on which to base the models. In order to address this need, an international team of epidemiological modellers were brought together to form the NTD Modelling Consortium. Members of the consortium were asked to provide quantitative analyses to support the NTD control efforts by

- validating current strategies,
- suggesting more impactful strategies,
- evaluating new tools as they arise from on-going studies,
- providing guidance on what the ‘end game’, beyond the 2020 goals, might look like.

Alongside this core project, the methods and models developed by members of the consortium have the potential to

- help countries understand whether they are on-track to WHO goals and, if not, how long and what strategies are needed to get there
- give countries guidance on when and how to best check on progress
- provide guidance on certification of elimination

There would also be opportunities for extending NTD models to include cost effectiveness and provide tools for policy at a local level, depending on the quality of the models and available data.

Importantly, for each of the diseases in this core research (Table 1), the research team includes two or three modelling groups per disease, to provide scientific robustness through investigating the same questions using a variety of approaches, mirroring other modelling consortia. The NTD Modelling Consortium is unusual amongst existing modelling consortia because it crosses a number of epidemiologically distinct infections, with different types of etiological agents and modes of transmission (Table 1). This diversity of diseases studied and the range of research groups and approaches allows the consortium to exploit similarities between diseases, such as vector-borne dynamics or the impact of mass drug administration (MDA), broadening the scientific base from which the analyses are motivated. In addition the research teams can work together to address common problems such as clarity on definitions and sharing good quality data. The group are also discussing different methodologies and techniques for model validation, testing and comparison.

The first analyses of these nine diseases by this research team has been presented as a collection in Parasites and Vectors (http://www.parasitesandvectors.com/series/ntdmodels2015) The analyses range from developing completely new models of diseases for which the epidemiology is still highly uncertain to bringing together models with a long history in order to achieve consensus on best strategies to achieve the 2020 goals. This paper reviews these results with the aims of

- Introducing the collection to non-modellers
- Introducing the collection to modellers from related fields
- Highlighting the key new policy insights
- Providing an overview across papers in the same disease
- Providing an overview across diseases

The main part of this paper takes the reader through the analyses disease by disease, starting with diseases that are being treated through preventive chemotherapy (PCT) (lymphatic filariasis, onchocerciasis, schistosomiasis, soil transmitted helminthiasis and trachoma) followed by the intensified disease management (IDM) diseases (Chagas disease, the Gambian form of human African trypanosomiasis, leprosy and visceral leishmaniasis in the Indian sub-continent). These disease-specific sections are followed by a discussion of general lessons learned and next steps.

### Preventive chemotherapy diseases

Preventive chemotherapy and transmission control (PCT) is the main strategy for control of onchocerciasis, lymphatic filariasis, schistosomiasis, soil-transmitted helminthiasis and trachoma. The strategy involves regular provision of preventive treatment (in the form of mass drug administration (MDA) campaigns) to entire populations or targeted risk groups (e.g., schoolchildren). This strategy reduces disease progression in treated individuals and prevents transmission of infection to others. Mass drug administration (MDA) programmes are rapidly expanding, although important questions remain. For example, will the planned MDA programmes be sufficient to achieve elimination in all epidemiological settings? To what extent is successful elimination jeopardized by low coverage and systematic non-adherence? When, and on the basis of what criteria, can MDA be safely interrupted [6]? Several of the modelling analyses highlight the importance of groups who systematically, or semi-systematically do not access MDA programs in sustaining transmission. This potential for undermining program success is particularly acute if groups of the population who are most at risk through their behaviours (e.g., those who most frequently go to the river) are also those who are most difficult to access through an MDA campaign. The results support previous analyses that increased coverage, across different age groups, or through general coverage, may be more important than frequency of treatment.

### Lymphatic filariasis

#### Background

Lymphatic filariasis (LF) is caused by a group of mosquito-borne filarial nematodes (*Wuchereria bancrofti* (responsible for 90 % of cases), *Brugia malayi* or *Brugia timori*) and can lead to chronic morbidity, such as lymphedema, which is associated with pain, severe disability and resulting social stigmatisation [7–9]. About 1.2 billion people are at risk of LF in tropical and subtropical countries in Africa, Asia, the Western Pacific, the Caribbean and South America. The Global Programme to Eliminate Lymphatic Filariasis (GPELF) was launched in 2000, aiming to eliminate the disease as a public health problem by 2020 by mass drug administration (MDA). In areas co-endemic with onchocerciasis, the combination of drugs used in MDA is ivermectin (IVM) and albendazole (ALB), whereas diethylcarbamazine (DEC) and ALB are used in other endemic regions. The current MDA strategy is to have yearly treatment at 65 % coverage of the total population for at least 5 years, followed by regular transmission assessments to identify whether transmission has been broken. Morbidity management will accompany initiation of MDA programmes.

A number of countries have reached the targets of stopping MDA and interrupting transmission, while others have scaled up their treatment programmes and are getting close to these targets, by reducing the risk of infection for hundreds of millions of people [10]. However, there are still large numbers of affected populations, who are predominantly in sub-Saharan Africa, and unlikely to receive the minimum 5 rounds of treatment by 2020. In such areas, adjusted strategies may be needed to accelerate elimination.

### Modelling approaches

Three distinct models have been used to evaluate the 2020 goals in a number of key settings [11–13]. All models capture heterogeneity in individuals’ exposure, whilst there exist differences in assumed acquired immunity and filarial worm biology. The model by Irvine et al. is an individual-based microsimulation. Model predictions were tested by fitting to the age-profile of infection in a survey prior to (Kenya) [14] and during an intervention (Sri Lanka) [15] and predicting forward the simulated microfilariae (mf) intensity distribution and prevalence in subsequent years was compared and found to be in good agreement to the data, but there were discrepancies in ICT prevalence.

Jambulingam et al. used the established individual-based, stochastic microsimulation model, LYMFASIM, taking into account variability in immunity, transmission potential and individual efficacy of MDA. The model was fitted to age-specific, longitudinal data describing the impact of integrated vector management on the intensity of *Wuchereria bancrofti* infection in Pondicherry, India [16].

Singh et al. [12] used a deterministic and age-structured model of genus-specific LF transmission. The model was calibrated using 22 pre-control settings from Africa, South East Asia and Papua New Guinea. Fitting was performed in a Bayesian melding framework to mf age-prevalence in a pre-control setting.

### Policy implications

**Fig. 1:**
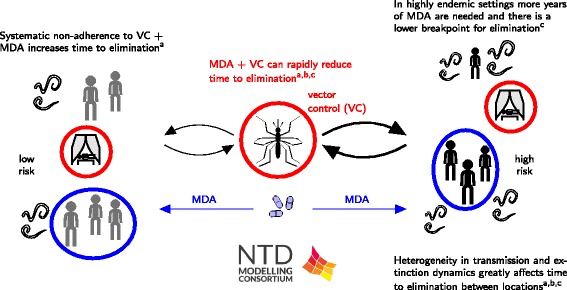
Schematic of LF results. The results include: a) highlighting that heterogeneity in human exposure and intervention greatly alters time to elimination by Irvine et al. [11]; b) a description of the association between antigenaemia and the presence of adult worms by Jambulinga et al. [13]; and c) a Bayesian fitting methodology of a deterministic model including information on model inputs and outputs by Singh et al. [12]

Irvine et al. identify a number of key areas that are important to address with regards to an elimination programme (Fig. 1a) [11]. Over a five-year timeline, twice-yearly annual MDA at 65 % coverage was found to be the most effective of all strategies considered. However, if twice-yearly MDA is not feasible, then an MDA programme combined with vector control (VC) can also have a similarly high probability of success in all settings. Annual MDA at 80 % coverage with no VC was found to be only effective in low and medium settings (less than 15 % mf prevalence) and annual MDA at 65 % coverage was found to be only effective for lower endemic settings (less than 10 % mf prevalence). A number of systematic adherence issues were found impact the success of a programme such as individuals who are not accessing the intervention also having higher risk of infection; use of long lasting insecticidal nets (LLINs) being correlated with adherence to MDA for an individual; and systematic compliance to MDA.

The model of Singh et al. indicates heterogeneity in local transmission and extinction dynamics vary greatly between settings (Fig. 1c) [12]. They showed that timelines to parasite elimination in response to the current MDA and various proposed MDA strategies with vector control also varied significantly between the study sites. Assessment of annual bite rates without the presence of vector control highlighted that very low prevalence is required to achieve true elimination because the subsequent probability of recrudescence is very high (between 69 and 100 %). Including VC, however, markedly reduces the duration of interventions required to achieve elimination as well as decreasing the risk of recrudescence.

Jambulingam et al. use their model to investigate the required duration of MDA to achieve elimination and to assess how low the microfilaraemia and antigenaemia prevalence must be to ensure elimination (Fig. 1b) [13]. The required number of treatment rounds for achieving elimination was found to depend strongly on local transmission conditions (reflected in baseline endemicity) and achieved coverage. For instance, in low endemic settings as few as 2 rounds might be sufficient if coverage is as high as 80 %, while annual MDA may have to continue for >10 years in high endemic areas if coverage is as low as 50 %. The study also shows that the critical thresholds used as endpoints for MDA, will be dependent on local transmission conditions: in low-transmission settings (low baseline endemicity) more residual infection may remain than in high-transmission settings (high baseline endemicity), because the relatively low biting rate in the former prevents resurgence of infection.

Although different modelling approaches were used, all models agree that timelines to LF elimination will depend on the epidemiological conditions and achieved coverage. These findings have important implications for on-going elimination programmes that should be taken into account in monitoring and evaluation. Transmission assessment surveys should ideally be targeted at the sites with the highest transmission intensity and lowest coverage: once elimination is achieved in these settings, it should also be achieved in other settings where conditions are more favourable for elimination.

#### Knowledge gaps and next steps

All three LF models have been fitted against mf prevalence data stratified by age. The use of mf and circulating filarial antigen (CFA) intensity measurements, where such studies are available, would greatly enhance the fit of the models to provide further insight into key underlying assumptions on exposure and immunity heterogeneity. A more direct comparison of the models for particular settings would further establish the systematic uncertainty between the models.

All three models need to be quantified and validated against disease prevalence by incorporating knowledge on disease dynamics and progression. This can help with setting new targets to reach the goal of LF elimination as a public health problem and identify aspects that need to be addressed to achieve this target. Models have to be made user friendly with minimum inputs/outputs for application in decision making and evaluation by programme managers [17].

### Onchocerciasis

#### Background

Human onchocerciasis is a disease caused by the filarial nematode *Onchocerca volvulus* and transmitted by blackfly vectors. Chronic infection can lead to skin disease, visual impairment and eventually blindness. It occurs primarily in tropical sub-Saharan Africa but some foci also exist in Yemen and Latin America. In recent decades, the disease burden of onchocerciasis has been greatly reduced by the Onchocerciasis Control Programme in West Africa (OCP, 1974–2002), the African Programme for Onchocerciasis Control (APOC, 1995–2015) and the Onchocerciasis Elimination Program for the Americas (OEPA, 1991-present).

In the Americas, OEPA has successfully interrupted transmission in most foci through 6- or 3-monthly mass drug administration (MDA) of ivermectin [18–23]. Annual or biannual ivermectin distribution has also eliminated onchocerciasis from several African foci [24, 25], although elsewhere transmission is on going despite prolonged MDA [26, 27]. In view of this evidence, the World Health Organization (WHO) set ambitious targets for the elimination of onchocerciasis, which is to be achieved by 2015 in the Americas and Yemen, by 2020 in selected African countries, and by 2025 in 80 % of African countries [3, 28].

#### Modelling approaches

The individual-based microsimulation model, ONCHOSIM [29, 30] and the population-based deterministic model EPIONCHO [31–33] have been developed independently at Erasmus MC and Imperial College London respectively.

A comparative modelling study is presented which explores the level of agreement between EPIONCHO and ONCHOSIM in estimates of the required durations to eliminate onchocerciasis. After harmonization of key input assumptions, predictions were made for a range of epidemiological settings (from mesoendemic to very highly hyperendemic or holoendemic) and programmatic (annual or biannual MDA at variable levels of population coverage) characteristics.

Simulation endpoints were defined by two criteria: (1) the duration of MDA required to reduce the mf prevalence below a threshold of 1.4 % (this is the provisional operational threshold for treatment interruption followed by surveillance (pOTTIS); and (2) the duration of MDA required to drive the parasite to local elimination. This was determined by reaching the transmission breakpoint in EPIONCHO and by a high (99 %) probability of stochastic fadeout in ONCHOSIM.

#### Policy implications

**Fig. 2:**
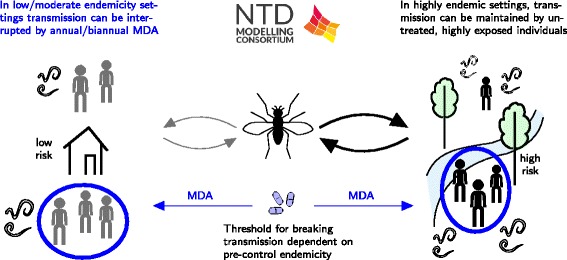
Schematic of onchocerciasis results. The results include a comparison of a stochastic individual-based model (ONCHOSIM) and a deterministic population-based model (EPIONCHO) and an investigation into the impact of systematic non-adherence in different endemicity settings by Stolk et al. [71]

Both EPIONCHO and ONCHOSIM indicate that elimination of onchocerciasis is feasible in mesoendemic settings by annual MDA with ivermectin alone (Fig. 2). The models’ predictions regarding the feasibility of elimination in settings with higher endemicity are more divergent, however, with ONCHOSIM being more optimistic than EPIONCHO. Both models agree that neither annual nor bi-annual MDA will achieve elimination in holo-endemic areas within a reasonable timeframe. Therefore, in highly endemic settings alternative intervention strategies should be considered.

More work is needed to validate the mf prevalence threshold used as endpoint for MDA. Results from the ONCHOSIM simulations indicate that, the 1.4 % threshold was too low for lower endemicity settings, resulting in unnecessary long continuation of MDA. The opposite is true at higher endemicity settings; the time required to reach the pOTTIS is shorter than the time required to drive the parasite population to elimination. In practice, the decision to stop is made for entire areas, with varying endemicity levels at baseline. The critical threshold should be set low enough to ensure elimination in the sites with highest transmission.

#### Knowledge gaps and next steps

Differences between EPIONCHO and ONCHOSIM in the projected infection dynamics and required durations to reach elimination will be further investigated to fully understand the strengths and weakness of the two contrasting modelling approaches. Ultimately a process of comparison, validation and refinement-followed by finescale locale projections will help to reach consensus on optimising intervention strategies to reach the global health communities’ elimination objectives across Africa. In order to perform these analyses, the researchers will require access to similar datasets from long-term programmes. Through testing both model predictions against these data, there can be increased confidence in the predictions on how altered strategies can be used to increase the probability of elimination.

### Schistosomiasis

#### Background

Schistosomiasis, or bilharzia, is caused by the adult worms and eggs of trematode flatworms of the genus *Schistosoma.* The adult worms live in the blood vessels where the females release eggs which are then passed out of the body in urine or faeces. In freshwater these eggs then infect snails, which subsequently release larvae which pass into the skin during contact with water. Eggs released in the body cause inflammation and scarring of internal organs, leading to negative developmental outcomes for children and adult pathology. Highest prevalence is seen in children, who are targeted for school-based deworming, which aims to control morbidity. At-risk adults are also often targeted, however, the target of eliminating transmission may require additional interventions, including water sanitation and hygiene (WASH) as well as snail control.

Current WHO guidelines define broad prevalence bands to indicate how school-age treatment should proceed. Models can be used to investigate the impact of this approach and update the guidelines to give them a stronger scientific underpinning. However, it is expected that current WHO control recommendations will need to be substantially revised based on the WHA shift toward 2020 elimination goals. The findings of present modelling efforts, and the use of further ad hoc model-based projections for different treatment scenarios, will be able to inform the development of the next generation of more evidence-based WHO policy recommendations for schistosomiasis control.

#### Modelling approaches

Modelling has been used to address many of the operational questions around frequency and needed coverage of schistosomiasis treatment, but until now, rarely been used to directly assess and predict the impact of PCT-MDA control programmes.

The basic aims were to fit two existing models to available detailed data for each parasite species, and to determine the likely long-term impact of current selective or MDA control programs to identify optimum antihelminthic treatment schedules to control schistosome infection. The models sought to define these schedules for low, medium and high transmission settings.

Two modelling approaches are proposed in the current issue: one of them employs *mean worm burden formulation* for age-structured populations [34], another one is based on *stratified worm burden* setup. Both modelling approaches incorporate the essential features of in-host biology, such as worm mating and density-dependent fecundity. The principal difference between models lies in their underlying assumptions: the hypothesized “negative binomial” worm burden distribution [35], and the assumption-free “dynamic” worm strata (with prescribed patterns of egg release) [36].

Anderson et al. [35] reconstructed the global trend in MDA coverage from the mean of national coverage data across endemic countries. This trend was then extended to estimate the likelihood of reaching the 2020 coverage target. These treatment estimates were then used to project changes in average worm burdens up to and beyond 2020.

Gurarie et al. [34] based their analysis on earlier calibrated models of Kenyan communities, and newer data sets from the SCORE study in Mozambique. The short term analysis assessed prevalence reduction under SCORE regimens through the year 2020. The long term analysis explored feasibility of specific target reduction over 30-year period under different control scenarios.

#### Policy implications

**Fig. 3:**
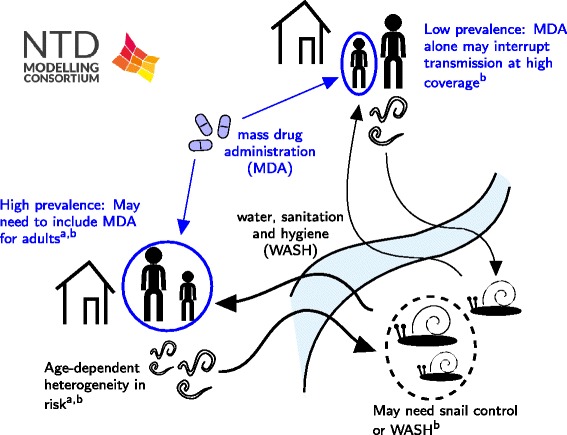
Schematic of schistosomiasis results. The results include: a) an assessment of the potential success of MDA in different scenarios using a deterministic modelling framework by Gurarie et al. [36]; and b) an investigation into the feasibility of elimination using an age-structured deterministic model by Anderson et al. [35]

Long-term control predictions of two model types did differ in several respects. Specifically, the key ingredients of this model, as employed in its analysis and simulations, follow MDA impact on basic reproduction number, R0, and whether transmission breakpoints (resulting from the underlying assumptions on worm distribution) can be reached. Anderson et al. thus predict that persistent long term MDA control can bring about elimination of *Schistosoma mansoni* transmission (Fig. 3b), but this was not the case for Gurarie et al. (Fig. 3a). The stratified worm burden systems in the model of Gurarie et al. suggest that breakpoints may not exist or they could be too low to be practically relevant (see [34]). An important implication of control analysis by Gurarie et al. is that MDA alone may not bring about elimination, or sustained low-level infection, even under moderate-to-low transmission intensity. Any successful end-game strategy will require additional interventions, including snail control, environmental and behavioral modifications related to exposure, sanitation, possibly with the aid of vaccines.

Anderson et al. predict that the current trend in MDA coverage, extrapolated to 2020, will have a major impact on *Schistosoma mansoni* burdens overall, with reductions of around 85 % by 2020 and elimination within the following decade in low transmission settings. Sensitivity analysis suggests that some coverage of adults is essential to achieve elimination but little is to be gained in transmission blocking by treating young children (pre-school aged children). However, higher levels of adult coverage show diminishing returns in terms of effectiveness.

Of note, the two groups’ models agreed regarding the need to achieve high levels of treatment coverage with more frequent drug delivery (at least annual) for best effect, particularly in high transmission settings. The ongoing research will elucidate some of these issues, including the value of mixed interventions, and help to further develop optimal control strategies.

#### Knowledge gaps and next steps

Results from validation against re-infection data suggest that other mechanisms are necessary to accurately reproduce the age profile of infection after treatment. A key difficulty is being able to resolve the influence of age-dependent force of infection and immune response mechanisms. Considerable inroads into the understanding of this complex area have already been made [37, 38]. Combining these approaches with high quality re-infection data should allow the contributions of different mechanisms to be more thoroughly teased out. However, an essential component will be the availability of high-quality longitudinal re-infection data, ideally at the individual level, which is proving difficult to obtain.

The interpretation of raw data is hampered by issues with current diagnostic techniques. Models of helminth transmission are based around representations of worm numbers within hosts, but the connection between worm burdens and the output of egg-counting diagnostic techniques, such as Kato-Katz, are not well characterised, although it is known that sensitivities can be quite low. Antigen and antibody based techniques promise more sensitive techniques, but lose the quantitative nature of the egg counts and will require careful calibration of the models [39].

The schistosomiasis researchers will continue to study the impact of school-based and community based interventions on both *S. mansoni* and *S. haematobium* through more detailed analysis of epidemiological studies, addressing the urgent need for these models to be tested against multiple settings. They will also consider the effect of WASH and snail control, where such data are available. The aim will be to provide guidance on which areas will need which interventions for control and elimination.

### Soil-transmitted helminthiasis

#### Background

Globally, over 1 billion people are infected with soil-transmitted helminths (STH). The three major STH species targeted for control are *Ascaris lumbricoides* (roundworm) and *Trichuris trichiura* (whipworm), both of which tend to exhibit highest prevalence and intensity amongst children, and hookworm (*Necator americanus* and *Ancyclostoma*), which tends to have highest prevalence and intensity amongst adults.

In recognition of the STH disease burden, the WHO has set the target of implementing annual or semi-annual MDA for pre-school- (preSAC) and school-aged children (SAC) and women of childbearing age (WCBA) in endemic areas with an overall coverage of at least 75 % by 2020. The associated parasitological goal is to achieve <1 % prevalence of moderate-to-heavy intensity infection in these target populations (and thus prevent most morbidity). However, given that current programs mostly target preSAC and SAC, the feasibility of controlling STH by 2020 with current strategies can be questioned, in particular for hookworm, which is most abundantly present in adults.

The WHO goals and treatment guidelines do not differentiate among the individual species that make up the STH group, but categorise the treatment approach primarily in terms of overall STH prevalence. In terms of life-cycle and natural history within the host, this is a reasonable assumption, although behaviour outside the host differs but it ignores the significant quantitative differences between species. Additionally, the guidelines only consider a narrow range of responses to STH prevalence (no treatment, annual or biannual treatment). This is motivated by a desire to directly and cost-effectively reduce morbidity in children, who are a key risk group. However, it ignores the possible long-term benefits of an approach that could reduce the contributions of the whole community to transmission, thereby leading to transmission break and cessation of annual or bi-annual treatment altogether.

The three species within STH have significant differences in age-intensity profiles, worm fecundity, and response to treatment. The qualitative range profiles indicate different distributions of worm burdens as well as different forces of infection by age for the three species. Further differences between species are indicated by large differences in worm burden and the characteristics of worm fecundity between species, as indicated by worm expulsion studies. A further key difference in the context of chemotherapeutic control strategies is the response of the three species to treatment with the standard anthelminthic drugs, albendazole and mebendazole: While these drugs are highly effective against *Ascaris* and, to some extent, hookworm, efficacy against *Trichuris* is much lower, which could have an effect on the choice of control strategy.

#### Modelling approaches

In this collection there are two models addressing control and elimination of the different soil-transmitted helminths. Coffeng et al. presented WORMSIM, an individual-based model for control by 2020 [40]. With WORMSIM, the researchers synthesised relevant available information on hookworm biology, and capture heterogeneities in transmission and MDA participation. The model predictions were compared to longitudinal parasitological data in WCBA from Vietnam spanning five years, collected pre-control and during PC. For varying levels of pre-control endemicity, the researchers predicted the impact of currently recommended MDA strategies, as well as the impact of more intense strategies (higher frequency and coverage of MDA), health education and improved access to WASH, and systematic non-participation of individuals in MDA programs.

The approach of Truscott et al. was to use a deterministic age-structured model to describe the dynamics of the parasites within the host population and the impact of increasing levels of MDA coverage [41]. Stochastic individual based models were also constructed by Truscott et al. but the average predictions were identical to the deterministic model and hence the main focus in their paper is on the deterministic outcomes. The same basic model structure is employed for each of the STH species, reflecting the very similar life-cycles of the three species, but the parameterization in each case is based on species-specific data taken from baseline age profiles and expulsion studies. As a result, the dynamics of the model in response to MDA is quite different for each species. The accuracy of the model in describing the evolution of worm burden under MDA was tested for Ascaris against longitudinal baseline and reinfection data. Model results are in broad agreement with the data, with some discrepancies in individual age groups. To drive the changes in worm burden up to and beyond 2020, a long-term trend in MDA coverage was constructed to drive control and, potentially, elimination of parasites. The trend was based on WHO records of average national coverage in SAC and pre-SAC in endemic countries, interpolated forward in time to meet the proposed 2020 goals or 75 % in SAC and pre-SAC. The data suggests that current trends in MDA coverage are approximately in line with reaching the stated goals by 2020. Both models employed in this study are amenable to the implementation of multiple forms of MDA, targeting multiple helminth species using different drugs. Detailed sensitivity analyses for parameter uncertainty were performed as were validation studies using reinfection data post chemotherapy using parameter estimates derived independently form the reinfection data.

#### Policy implications

**Fig. 4:**
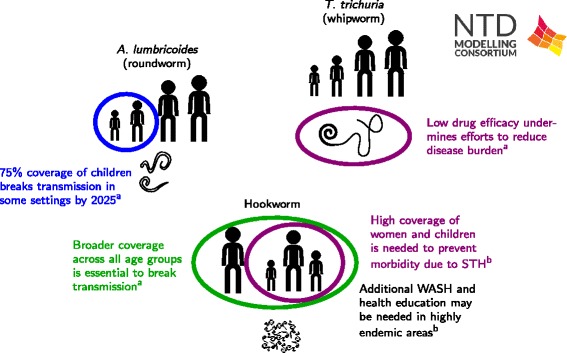
Schematic of STH results. The schematic includes results from: a) a deterministic transmission model by Truscott et al. applied to Ascaris, Trichuris and hookworm [41]; and b) a stochastic, individual based model of hookworm transmission by Coffeng et al. [40]

The predictions by WORMSIM [40] confirm that to achieve control of hookworm morbidity, women of childbearing age have to be targeted with PC (Fig. 4b). Furthermore, Coffeng et al. conclude that to achieve control in highly endemic areas, the drug albendazole should be preferred over mebendazole, and possibly additional interventions such as health education and improved access to WASH are needed (Fig. 4a). They also illustrate how systematic non-participation to PC undermines programme effectiveness, even during high-frequency PC.

Results from Truscott et al. [41] show that the impact of the recent and planned increases in MDA coverage will depend strongly on species. For Ascaris, worm burden across the host population is reduced by 70 % by 2020, leading to elimination within the following decade if coverage levels are maintained. The reduced efficacy of albendazole against Trichuris mitigates the effect of treatment against the species, achieving only a 44 % reduction in worm burden with no possibility of elimination with continued target levels of coverage. For hookworm, the MDA is even less effective, due to the bulk of worm burden (>70 %) being in adults who are outside the treatment regime.

The implications are that the treatment response to STH needs to be tuned to reflect the dominant species in a given area. Where that species is Trichuris or hookworm, approaches beyond treatment of SAC may need to be considered, particularly where transmission is high. For hookworm, some degree of treatment of adults will be necessary to significantly reduce burden or achieve elimination. For Trichuris, a higher efficacy drug or more frequent treatment could potentially be highly effective at reducing worm burden.

#### Knowledge gaps and next steps

As for schistosomiasis (above), the predictions of the impact of age-based deworming programmes depend on the assumptions of the contribution of different age groups to transmission and of acquisition of infection through a shared exposure to the ‘infective pool’. They also highlight the challenges of interpreting Kato Katz, although, unlike schistosomiasis, historic studies of the relationship between egg output and adult worm burden make the problem slightly less acute.

Next steps for these groups are to extend their model validation to more species and multiple settings, and to do a more systematic model comparison of their predictions to quantitative guidance on thresholds for different treatment coverage.

### Trachoma

#### Background

Trachoma remains the world’s leading cause of infectious blindness [42]. Repeated ocular infection with the bacterium *Chlamydia trachomatis* leads to episodes of conjunctival inflammation. With repeated infection this inflammation can progress to scarring. The resultant scarring leads to the in-turning of eyelashes, known as trachomatous trichiasis (TT) which abrade the corneal surface of the eye, ultimately resulting in blindness [43]. It is currently estimated that 84 million individuals are living with active disease, where the highest burden of infection is concentrated in young children [42]. In addition, 1.2 million people are estimated to be blind as a result of infection [42]. While there has been some success at controlling trachoma infection, it remains endemic in 50 countries.

The WHO aims to control infection and eliminate trachoma as a public health problem by 2020 [43]. To help achieve this, the WHO supports the implementation of the SAFE strategy: Surgery for trichiasis, Antibiotics for treatment, and Facial cleanliness and Environmental improvements to reduce the probability of transmission [43]. Effective control relies on the successful implementation of antibiotic treatment as well as long term reductions in the overall level of transmission. The decision to declare that trachoma has been controlled within a community or whether or not further antibiotic treatment is required is based on the prevalence of trachomatous inflammation–follicular (TF) in children aged 1–9 years [43]. However, it is possible that other surveillance data sources, such as trachomatous inflammation-intense (TI) prevalence or the detection of active chlamydial infection through PCR may provide additional information on the dynamics of transmission within the population [44]. This can help to assess whether sustained control is being achieved or whether infection is remerging.

#### Modelling approaches

Two distinct models were developed to address two key areas in trachoma transmission control and surveillance. The developed model by Gambhir and Pinsent [45] was a deterministic susceptible, infected, susceptible (SIS) transmission model, which was age-structured and followed individuals from their first infection to their last (a ‘ladder of infection’), and accounted for the development of immunity within the population as the number of infections experienced increased. This model assessed the impact of multiple annual rounds of MDA and the implementation of F and E on the long-term transmission dynamics of infection, within three different transmission settings. In addition, the short and medium-term impact on the effective reproduction number, *R*_e_, was also assessed within each transmission setting, as a measure of the potential for post-treatment infection rebound.

Liu et al. based their model on a stochastic SIS process [44]. The model was a hidden Markov process of infection at the community level, and numerical evaluation of the Kolmogorov forward equations enabled straightforward likelihood fitting based on clinical trial data from the Niger arm of the Partnership for the Rapid Elimination of Trachoma (PRET) study. Model fitting utilized several observations, including PCR data, the clinical sign TF, and the clinical sign TI. Because TF guides policy and intervention, we produced forecasts of future observations of TF, thereby evaluating model predictions on a test set separate from the training set. Both TI and laboratory infection tests led to moderate, but not significant, improvement in forecasting the future level of infection within the community and that including a delay in TF recovery improves forecasting.

#### Policy implications

**Fig. 5:**
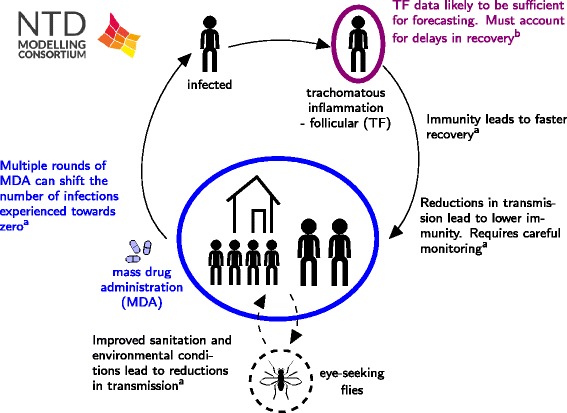
Schematic of trachoma results. The schematic includes results from: a) a transmission model including consideration of immunity by Gambhir et al. [45]; and b) a statistical analysis of the most informative data for forecasting trends in prevalence by Liu et al. [44]

Gambhir et al. suggest that a combination of MDA and reductions in the overall level of transmission within both high and low transmission communities would ensure that long-term control of transmission could be achieved (Fig. 5a). These control measures result in the overall number of infections experienced by an individual in the community at any point in time becoming lower than prior to the introduction of the interventions. However, the rapid and dramatic reductions in transmission that may occur due to these interventions may result in a slower acquisition of immunity to infection. This may mean that although individuals are getting infected less frequently, when they do they have a higher infectivity and are infectious for longer. To monitor these potentially adverse outcomes it may be important to collect infection samples from a sub-section of the adult population, as well as young children to ensure that reductions in population level immunity are not occurring.

Liu et al. designed a model to assess which data sources are more informative for predicting the future state of infection in a community (Fig. 5b). They suggested that TF data alone was just as informative for forecasting the future level of infection in the community as when TF, TI and PCR data were combined. If applied to data from particular settings, the model can be used to determine which regions are likely to attain the goals, and if not which additional interventions may be necessary in order to achieve them. If regions are identified as requiring fewer resources than anticipated, these resources could be moved to areas less likely to attain goals.

#### Knowledge gaps and next steps

A number of different model structures need to be compared and statistically validated, to assess which is the most suitable structure going forwards. For example, is an SIS model without age structure sufficient to capture the overall observed dynamics of infection? While individuals are no longer PCR positive, but are still TF positive, is it possible for them to become re-infected at this point? High resolution data will also help to disentangle the relationship and the time spent PCR and TF positive, and help with the explicit modelling of both of these stages. In addition, more longitudinal data will help to assess trends in transmission over time that have occurred as a result of different interventions. Much about trachoma remains poorly understood, and will probably remain unknown as we eradicate the disease. Models need to be validated and calibrated in collaboration with the International Trachoma Initiative (ITI) to make more global projections on the feasibility of the 2020 goals and where additional resources may or may not be needed. Yet for any model, an argument can be made that something, possibly important, should be added to it; validation through prediction can, in large part, resolve such issues - telling us whether our models are adequate to guide elimination campaigns.

## Intensified disease management diseases

A number of neglected tropical diseases are controlled by increased diagnosis and management of cases (intensified disease management, IDM). The four IDM diseases in this study are Chagas disease, the Gambian form of human African trypanosomiasis, leprosy globally and visceral leishmaniasis on the Indian sub-continent. Whilst these diseases cause significant morbidity and mortality, the disease courses are quite long, the epidemic growth rate is slow and the transmission is usually highly focal. They are often associated with disadvantaged populations and hard-to-reach groups. Given this concentration of disease in populations with poor access to care, and the potentially long time periods over which their disease course and dynamics occur, these diseases have been difficult to study and so quantitative estimates of key parameters are scarce. In the model analyses of these diseases the authors have aimed to provide novel estimates of key parameters and provide both qualitative and quantitative insights on the dynamics of these infections and their consequences for control.

### Chagas disease

#### Background

Chagas disease (etiological agent *Trypanosoma cruzi)* is the most important zoonotic vector-borne disease in the Americas, with an estimated 8 million people infected, ten thousand deaths per year and a disease burden, as estimated by Disability-Adjusted Life Years (DALYs), of 7.5 times that of malaria [46]. Chagas disease is endemic in Latin America, and has been steadily spreading to other parts of the world including North America, Europe, and Australia [47]. Estimates suggest that over 8 million people are infected, but since many cases go undetected, the actual number of infections may be higher. A study estimated the global annual burden to be $627 · 46 million in healthcare costs and 806,170 DALYs [48]. However, since Chagas can result in chronic heart disease after years of being asymptomatic [46, 47], much of the costs of Chagas disease occur years into the future. Therefore, currently infected individuals are expected to cost $7 · 19 billion per year and $188 · 80 billion throughout their lifetimes [48]. Transmission mainly occurs via the triatomine bug [47] (also known as the “kissing bug”), which can acquire the *T. cruzi* parasite by taking a blood meal from an infected mammal. Transmission from vector to human occurs when a *T. cruzi-* infected triatomine defecates (usually during or immediately after taking a blood meal) on an uninfected human, depositing the parasite on the skin. The bitten person often facilitates the parasite entering the bloodstream by rubbing or scratching the bite area and smearing the bug faeces into the bite or other areas with ready access to the bloodstream such as the eyes or mouth. Less frequently, transmission can occur through blood transfusion, congenital transmission (from infected mother to fetus), and organ donation [47]. Transmission can also occur orally through the ingestion of food contaminated with infected triatomine bug faeces and laboratory accidents [47]. Currently the main Chagas disease control methods are triatomine bug control, protecting food from contamination, and screening blood and organs for *T. cruzi*. Vector control methods include insecticide spraying, bed nets, and fixing the cracks in buildings (e.g., improved housing). Vaccines and other medications are under development [49–51].

The 2020 goals call for the interruption or reduction of transmission across all routes, and an increase in the number of patients under treatment. A major challenge in achieving these goals is not what to do, but how to do it on a wide enough scale to reach a sufficient proportion of those infected or at risk. The two strategies for interrupting vector-borne *T. cruzi* transmission are spraying indoor residual insecticides (IRS) and housing improvements. IRS must be applied on a regular basis to avoid re-infestation, and this has led to insecticide resistance in some triatomine species. Housing improvements can be effective, but they are disruptive and expensive. Thus, a central question is how often and for how long must these strategies be carried out to eliminate transmission, and which factors in the transmission scenario affect these efforts?

#### Modelling approaches

The modelling approach of Peterson et al. [52] was to examine the effect of synanthropic animals on *T. cruzi* transmission and prevalence in humans, and how animal presence affects the efficacy of vector control. Animals are important to consider because in most Chagas-endemic settings, there are numerous pets, livestock, and vermin that not only serve as food sources for triatomine bugs, but are also competent *T. cruzi* hosts. Thus an important question is whether it is necessary to target animals for Chagas control, as the current strategies only target the vector.

**Fig. 6:**
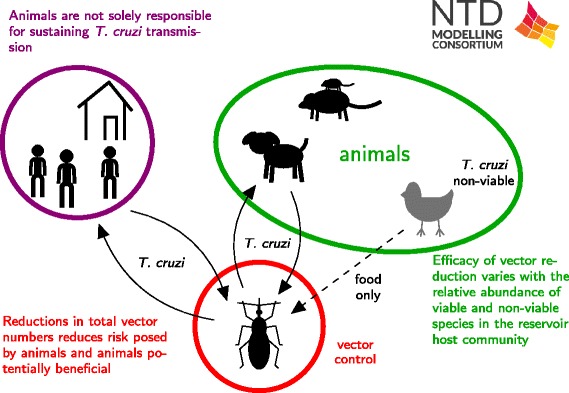
Schematic of Chagas results. The schematic describes a new transmission model for Chagas disease used to analyse the consequences of varying standard assumptions about the transmission cycle by Peterson et al. [52]

Peterson et al. focused their efforts on using models to test hypotheses on human-vector-animal interactions. This qualitative analysis showed that it is likely that animals do amplify transmission to humans in the absence of any vector control measures, because of their role as additional food sources for the bugs leads to increases in the vector population size (Fig. 6). However, if vector control measures are carried out that prevent the vector population from growing in the presence of animals, then animals can have a beneficial effect, even without reducing the vector population to zero, due to “diluting” the bites of the remaining vectors. This effect is then intensified if the animals are only food sources for the bugs and not competent *T. cruzi* hosts, which is the case for poultry or any other bird species.

#### Policy implications

These analyses highlight the importance of applying vector control to reduce total vector numbers, rather than temporarily reducing vector biting on humans. In different epidemiological settings, the most appropriate vector control method may be different. In particular, the availability of alternative animal populations for food sources which will enable the triatomine bugs to recover rapidly following spraying, can undermine the control efforts. These results also highlight the importance of entomological studies in endemic areas to understand the biting patterns of the triatomine bugs and how these are affected by changing densities of humans and other animals.

#### Knowledge gaps and next steps

A number of substantial knowledge gaps still exist regarding the transmission dynamics of Chagas disease, its prevalence and incidence in many countries, the potential points of intervention, the best ways to diagnose, monitor, and treat Chagas disease, and the impact and value of different control measures. Modelling efforts can help address these important gaps and guide current and future data collection efforts and intervention development and testing. An example of a subsequent modelling effort is to extend an initial model that allowed an investigation of how animals impact the force of infection. The researchers now plan to in parallel develop the transmission models and to use other data to inform the models on the force of infection by age and the basic reproduction number, *R*_*0.*_ An important source of information on the dynamics of Chagas in different areas will be age prevalence data from a variety of settings. Some of these data are prior to any form of intervention, which should allow estimation of the basic reproductive number. The availability of both pre- and post- intervention serologies will allow estimation of the impact of control measures and the additional efforts required to break transmission to humans. By estimating the force of infection for different regions and municipalities, researchers can examine the scale of the problem in a truly comparable way across Chagas-endemic areas.

### Human African trypanosomiasis, Gambian form

#### Background

Human African trypanosomiasis (HAT) is a parasitic vector-borne disease spread by tsetse (*Glossina* spp) and is fatal without treatment. There are two distinct forms, Rhodesian and Gambian HAT, with the Gambian form endemic in West and Central Africa and responsible for almost all (>95 %) HAT cases. Efforts to control the disease have led to a large reduction in the burden of disease, with reported cases falling from around 38,000 in 1998 to less than 4000 in 2014 [53]. Consequently, it is now targeted for elimination as a public health problem, defined as less than 1 case per 10,000 people per year, in 90 % of endemic foci by 2020 [54]. There are two stages of HAT disease and treatment is stage-specific.

Three main methods of intervention can be used in HAT-endemic areas:

1. Those infected with HAT will usually seek treatment by self-presentation at medical facilities when symptoms worsen, although this may not be until stage 2 disease.
2. Many endemic areas have active/mass screening campaigns to detect and treat both stage 1 and 2 cases.
3. Vector control using tsetse targets has been shown to substantially reduce tsetse population sizes [54]. However, vector control is not currently used in all endemic areas.

#### Modelling approaches

**Fig. 7:**
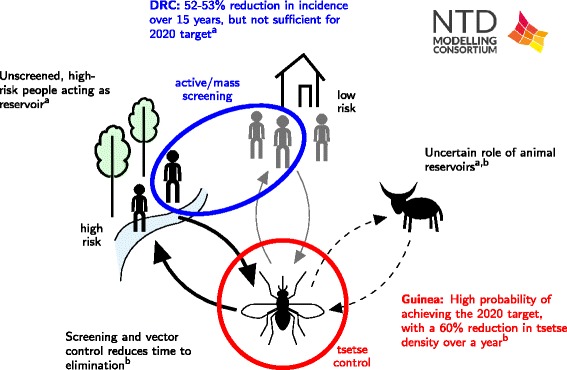
Schematic of HAT results. The results include a) quantitative estimates of the level of heterogeneity in human exposure and screening participation by Rock et al. [56]; and b) an assessment of strategies combining both human screening and tsetse control by Pandey et al. [55]

In recent analyses, two research groups have independently addressed the feasibility of the WHO goal of elimination as a public health problem by 2020 under current strategies using mechanistic mathematical models [55, 56]. Both models used differential equations to quantify stage 1 and 2 disease in humans, tsetse infection and possible animal reservoirs (Fig. 7). Pandey et al. also capture possible human population-level heterogeneity in exposure to tsetse bites and participation in screening.

The model of Pandey et al. was fitted to 2008–13 prevalence data from humans, non-human animals and tsetse within the Boffa HAT focus in Guinea, where mass screening and treatment have been combined with vector control (Fig. 7b) [54]. Annual tsetse control using tiny tsetse targets is modelled using a function which reflects a decline in their effectiveness throughout the year. Fitting of the model to the trial data was used to estimate key parameters on the underlying level of transmission and the impact of vector control measures. The calibrated model was used to estimate the achievability of the 2020 goal under the scenarios of vector control alone, or vector control combined with biennial or annual screening under 2013 coverage levels. The model’s projections accounted for the impact of the 2014–5 Ebola epidemic on HAT control efforts.

In a related approach, Rock et al. used data from two health zones, Yasa-Bonga and Mosango, in Bandundu province of the Democratic Republic of Congo (DRC), one of the highest incident areas of Gambian HAT (Fig. 7a). Bandundu has screening campaigns, but, partly due to its size, has not yet implemented a vector control programme. The model was fitted to 13 years of case data to estimate the underlying levels of transmission and the effectiveness of current screening campaigns. The expected time to elimination as a public health problem was predicted for a range of hypotheses for human heterogeneity under two levels of active screening: the highest level achieved (in 2009); and the mean level observed between 2000 and 2012.

#### Policy implications

Each modelling study included an analysis of the achievability of the 2020 goals in the setting analysed. Pandey et al. predict that annual implementation of vector control, at the same level attained in 2013, has at least a 77 % probability of eliminating HAT as a public health problem in Boffa by 2020. If biennial screening or annual screening is conducted alongside vector control then the probability of elimination by 2020 increases to over 90 %.

While there is evidence that active screening and treatment in Yasa-Bonga and Mosango have led to a 52–53 % reduction in new infections over 15 years, Rock et al. predict that the region is unlikely to meet the elimination goal until 2059–2091 under the highest level of current active detection and treatment. Incorporating human heterogeneity in the model improves the fit to observed data; the best model fit is obtained when humans who are more exposed to tsetse bites are assumed to never participate in active screening. Results suggest that current active screening campaigns could be further improved by targeting high-risk individuals and those who have previously not taken part in screening.

#### Knowledge gaps and next steps

Neither of these analyses were able to rule out the possibility of an animal reservoir for infection due to the nature of the available data. Pandey et al’s analysis suggests that vector control is efficacious irrespective of a reservoir, but in the presence of a reservoir, intervention strategies must be maintained, even after elimination, to prevent HAT re-emerging as a public health problem. Future modelling work utilising data on trypanosome prevalence in animals and tsetse host preference should enable better determination of the role of animals in disease transmission.

The modelling results highlight the level of geographic heterogeneity of HAT burden and the variety of intervention strategies currently used. Whilst some areas, such as Boffa, are on track to meet the 2020 goal, other regions may need to strengthen their existing strategies with complementary measures. In particular, Yasa-Bonga and Mosango are hard-to-reach regions with high incidence. Consequently, they are likely to be amongst the hardest areas in which to achieve elimination.

Moving forward it will also be important to examine how spatial heterogeneity in both transmission and interventions at a local level may impact disease incidence within a larger geographical area. To achieve this it will be crucial to have good estimates of demography, population sizes and, ideally, movements at a local level to inform models which include analyses of the spatial distribution of cases.

### Leprosy

#### Background

Leprosy, or Hansen’s disease, is an infectious disease caused by the bacterium *Mycobacterium leprae.* Transmission is believed to occur through close contact with an infected person, but the route of transmission remains largely undefined, and it would appear that only a small proportion of people that are exposed will eventually develop the disease [57]. Leprosy is diagnosed based on clinical signs and treated with multidrug therapy (MDT). Leprosy control rests on early diagnosis and treatment, which is thought to prevent both transmission and progression to leprosy-related disability.

Worldwide, more than 200,000 new leprosy cases are detected and reported annually from 121 countries [58]. This number has been fairly stable in the past 8 years, suggestive of ongoing transmission. Together, India, Brazil and Indonesia account for 81 % of all new cases, and only 13 countries reported more than 1000 new cases in 2014. Recently, WHO has formulated ‘roadmap targets’ for leprosy [3]. The targets set for the period 2015–2020, are: (1) global interruption of transmission or elimination by 2020, and (2) reduction of grade-2 disabilities in newly detected cases to below 1 per million population at global level by 2020.

#### Modelling approaches

The three analyses in the collection use distinct modelling and statistical approaches to assess progress of leprosy control programmes in different settings. Blok et al. [59] used a stochastic individual-based model SIMCOLEP to assess the feasibility of achieving global elimination of leprosy by 2020. SIMCOLEP simulates the life histories of individuals, the natural history of infection with *M. leprae*, and the transmission of leprosy in a population structured in households. Leprosy control includes passive detection and treatment. Household members of a detected case can be subjected to contact tracing. The model was fitted to the leprosy situation, including control, in India, Brazil and Indonesia on national and sub-national levels using data from the National Leprosy Elimination Program (India), SINAN database (Brazil), and Netherlands Leprosy Relief (Indonesia). Using the fitted model, future projections were made of the leprosy incidence, assuming continuation of leprosy control programs.

Linear mixed-effects regression models were used by Brook [60] to investigate the relationship between leprosy case detection rate at the district level and several state-level regressors: the incidence of tuberculosis, BCG vaccination coverage, the fraction of cases exhibiting grade 2 disability at diagnosis, the fraction of cases in children, and the fraction of cases which were multibacillary. Districts reported to have been targeted for enhanced case finding showed evidence of an increase in case detection. However, substantial unexplained differences between districts were seen (both in terms of new case detection rate and trend). Moreover, the overall rate of decrease was very small, controlling for the enhanced case finding.

Crump and Medley [61] developed a back-calculation approach to investigate the infection dynamics of leprosy. The model allows for varying effort or effectiveness of diagnosis in different time periods. Publicly available data from Thailand were used to demonstrate the results that can be obtained as the incidence of diagnosed cases falls [62]. Estimates of the incidence of new infections and clinical cases were obtained by year, as well as estimates of diagnostic efficacy. The method also provides short-term forecasting of new case detection by disease type, including disability status.

#### Policy implications

**Fig. 8:**
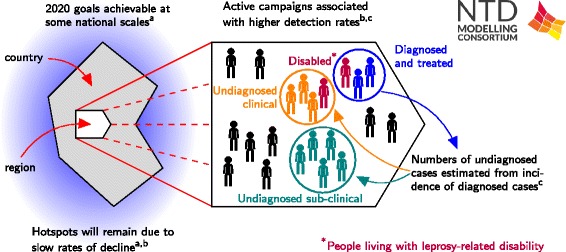
Schematic of leprosy results. The results include: a) a transmission model fitted to national and regional data from India, Brazil and Indonesia to predict future trends in leprosy incidence by Blok et al. [59]; b) statistical modelling of regional case detection data from India by Brook et al. [60]; and c) a back-calculation method to investigate underlying infection dynamics and predict future incidence by Crump and Medley [61]

Blok et al. showed that although elimination at national level is predicted by 2020, leprosy will still remain a problem at sub-national level (Fig. 8a). These high-endemic regions have multi-million populations in which rapid progress of leprosy control, even if conducted optimally, will not be achieved soon. The authors conclude that ongoing transmission of *M. leprae* will make global elimination of leprosy unlikely by 2020. Further control measures are needed to achieve the goals [59].

The analysis of new case detection rates from India by Brook et al. suggests an endemic disease in very slow decline, with heterogeneity across state and district levels (Fig. 8b). Active case finding was associated with a higher case detection rate, but not rapid leprosy control. Finer geographic resolution would improve analysis and bolster evidence-based policy assessment. Objective surveys may have a role to play in leprosy program evaluation, in view of differences in case reporting and in active case finding efforts.

Crump and Medley found that Bayesian back-calculation shows great potential to provide estimates of numbers of individuals in health/infection states that are as yet undiagnosed (Fig. 8c). This has the potential to provide valuable information for those managing or evaluating control programmes. The methodology is driven by available data, and provides an impetus for better reporting in that results can be quickly fed back to programs.

#### Knowledge gaps and next steps

There is relatively little known about leprosy with any degree of certainty. The long delay between infection and disease means that current diagnoses are a poor measure of current infection. Further modelling work may help to address this and also highlight areas where data collection would be valuable.

Blok et al. plan to include grade 2 disabilities and consider intervention programmes targeting contacts of leprosy patients; such as chemoprophylaxis, immunoprophylaxis and an anticipated diagnostic test for sub-clinical cases. Brook et al. plan to use their statistical modelling to inform a stochastic model to explore the use of targeted surveys and the effect of sustained active case detection. The back-calculation model of Crump and Medley will be further developed to consider gender and age. The three groups will be working with national and regional data of variable endemicity.

### Visceral Leishmaniasis

#### Background

Visceral leishmaniasis (VL) is caused by chronic infection with protozoan *Leishmania* parasites and is spread by infected sandflies. Annually, more than 80 % of the 200,000–400,000 global cases of symptomatic disease, and an estimated 15,000–30,000 deaths occur on the Indian sub-continent (ISC) [63]. There, VL is caused by *Leishmania donovani,* is spread by a single sandfly species, *Phlebotomus argentipes*, and is considered to be solely anthroponotic. VL, also known as kala-azar (KA), has been targeted by the WHO for elimination as a public health problem on the ISC, defined as less than 1 new case per 10,000 people per year at sub-district level, by 2017. Existing interventions focus on reducing transmission, mainly by reducing vector population densities through indoor residual spraying (IRS) with long-lasting insecticides (DDT and synthetic pyrethroids) and prompt diagnosis and treatment.

Individuals that develop KA, show symptoms of prolonged fever, anaemia, weight loss and spleen and liver enlargement, and usually die without treatment. Most individuals recover following successful treatment, though a small proportion (2–10 % on the ISC) go on to develop post-kala-azar dermal leishmaniasis (PKDL), a non-fatal dermatological condition characterised by a nodular or papular skin rash. However, the majority of individuals infected with the parasite are asymptomatic, but may be infected for many years; it is unclear if individuals ever completely lose infection and how long immunity lasts for those who develop it.

#### Modelling approaches

To address the question of whether the 2017 VL elimination target can be met with current interventions, it is necessary to obtain robust estimates of key epidemiological parameters and to assess how uncertainties in transmission affect the efficacy of different interventions. These issues have been tackled in separate analyses by two research teams [64, 65].

Chapman et al. [65] used statistical analyses to assess the risk of progression to KA based on serology test results, and a probabilistic model to estimate key parameters in the natural history of VL. Their model is fitted to data from a detailed epidemiological study conducted in three highly endemic villages in Bangladesh between 2002 and 2004, at which time no control interventions other than antimonial treatment and untreated bed net use were in place in the region. By fitting to the annual serology (rK39 antibody and leishmanin skin test) test results and KA onset and treatment dates from the study, the researchers estimate the duration of asymptomatic infection, the duration of immunity and the proportion of asymptomatic individuals that progress to KA.

Le Rutte et al. [66] describe the quantification of VL transmission between humans and sandflies on the ISC with 3 deterministic age-structured models. The principal source of infection to sandflies remains unknown, and Le Rutte et al. test three hypotheses for the source in their models - namely (1) asymptomatic infections, (2) re-activation of infection after recovery from initial infection, or (3) PKDL. All 3 models are parameterised with age-structured data from the KalaNet study, which consists of annual prevalence of infection (PCR), detectable immune responses (DAT) and incidence of VL in highly endemic clusters in India and Nepal as well as the percentage prevalence of infected sandflies in Nepal. The inclusion of age-structure in the models allows for detailed fitting and age-related heterogeneity in sandfly exposure. With these models they predict the impact of current interventions on VL incidence to estimate the feasibility of achieving the 2017 elimination target for the ISC. Predictions are made for three levels of VL endemicity and for optimal and sub-optimal IRS effectiveness, which may vary due to quality of implementation and vector resistance to DDT.

#### Policy implications

**Fig. 9:**
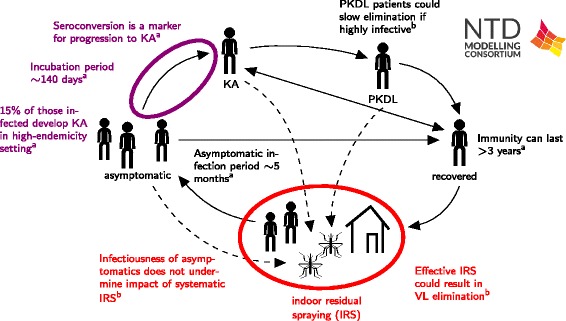
Schematic of VL results. The results include: a) new estimates of epidemiological parameters by Chapman et al. [64]; and b). a qualitative investigation of the impact of different life history assumptions on transmission dynamics and intervention efficacy by Le Rutte et al. [65]

The statistical analyses by Chapman et al. show that individuals who initially have high antibody levels are more likely to progress to KA than individuals with low or moderate antibody levels, and that those who seroconvert to high antibody levels have an even higher chance of developing KA (Fig. 9a). These findings suggest that individuals at high risk of progressing could be identified by screening, so that their infectious period and onward transmission could be reduced with improved access to treatment and targeted IRS. The fitting of the probabilistic model to the data gave estimates of 147 days (95 % CI 130–166 days) for the average duration of asymptomatic infection and 14.7 % (95 % CI 12.6–20.0 %) for the proportion of asymptomatic individuals progressing to KA - much longer and higher estimates than those reported previously [66], suggesting that asymptomatic individuals may contribute significantly to transmission.

The models of Le Rutte et al. show that the predicted impact of IRS differs per model variant, depending on whether asymptomatics, re-activated infections or PKDL cases constitute the main reservoir of infection (Fig. 9b). Further, the feasibility of achieving elimination of VL on the ISC strongly depends on pre-IRS endemicity and the effectiveness of IRS itself. Based on the assumption that cases of asymptomatic infection are the main reservoir (due to high numbers, and despite low infectivity towards the sandfly) and IRS is implemented optimally, the authors predict that VL may be eliminated in low and moderately endemic areas within six years of the start of IRS. For highly endemic areas and areas with sub-optimal IRS, additional interventions may be required.

#### Knowledge gaps and next steps

The relative infectivity of individuals in different disease stages is currently not known and thus neither is their contribution to transmission. Ongoing xenodiagnostic studies and additional longitudinal data on the prevalence of infection in sandflies during interventions will inform the transmission models regarding the most likely reservoir of infection, and enable the implementation of an appropriate model structure in an individual-based model by Le Rutte et al. In regions where it is predicted that the target of <1 VL case per 10,000 capita will not be reached, additional interventions may be required; the impact of these (such as a potential vaccine) will be explored by Le Rutte et al.

To aid estimation of the relative contributions of different disease groups to transmission, spatial and temporal variation in VL risk will be included in the probabilistic model of Chapman et al. Fitting this model to other longitudinal datasets will provide more robust estimates of the different disease stage durations and proportion of individuals progressing to disease, and an indication of the extent to which these parameters depend on endemicity and other risk factors. This work will be used to inform the development of future transmission models of VL for assessing the efficacy of different interventions.

### Discussion

The publications in this collection bring together a variety of different approaches to provide novel quantitative analyses that can inform policy development on the control and elimination of nine NTDs. For the PCT diseases existing and novel models have been brought together to assess the impact of current strategies, identify areas where they need to be adjusted and provide consensus insights on likely coverage needs and program duration (Table 2). For the IDM diseases, new models and methods have been developed and key parameters (such as the incubation period or proportion of infections accessing care) have been estimated (Table 3). In both areas, these are important steps forward. These analyses also identify the need for further work, as well as more rigorous model comparison and testing against more extensive datasets. Across the diseases, there are a number of common themes that emerge:

**Table 2:** Summary of modelling techniques used, PCT diseases

Paper		Model	Fitted to data from:	Predictions tested?	Technical advances	Model accounts for			Next steps
						Vector/environment dynamics	Heterogeneity in risk	Access to interventions	
Lymphatic filariasis									
Irvine et al.		Stochastic individual	Kenya and Sri Lanka	Yes	Heterogeneity in transmission and extinction dynamics greatly affects time to elimination	Deterministic vector dynamics. Single pool of vectors	Gamma distributed risk in exposure	Spectrum of access to repeat rounds of MDA and vector control, with cross-correlations with risk	Fit the model to intervention data and understand transmission dynamics at low densities
Jambulingam et al.		Stochastic individual	35 villages in India.	Yes	Association between antigenaemia and presence of adult worms.	Deterministic vector dynamics. single pool of vectors	Age dependent, gamma distributed exposure	Individual’s treatment compliance is semi-systematic	Estimating vector infection thresholds. Estimating probability of elimination
Singh et al.		Deterministic	Data from 22 villages from Africa, South East Asia, and Papua New Guine	No	Bayesian fitting including information about model inputs and outputs	Deterministic vector dynamics. single pool of vectors	Negative binomial distribution of worms	Random	Estimating thresholds for true elimination. Further understanding of parameter uncertainty
Onchocerciasis									
Stolk et al.	ONCHOSIM	Stochastic	Cameroon	Yes	Bringing the two models together and understanding differences in predictions	Deterministic vector dynamics. Single pool of vectors	Age dependent, gamma distributed risk	Age-dependent probability of receiving treatment. Lifelong compliance factor	The two models give different Mf intensities and prevalences after MDA, which needs to be investigated further
	EPIONCHO	Deterministic	Cameroon	Yes		Single vector compartment	Age- and sex-specific exposure to blackfly	Compliant and non-compliant groups	
Schistosomiasis									
Anderson et al.		Deterministic	Kenya	Yes	Using an age-structured model to assess the feasibility of elimination, and comparing model predictions to reinfection data	Environmental reservoir, constant decay rate. No explicit consideration of snail dynamics	Variability in exposure as a function of age. Negative binomial distribution of worms	Treatment reduced worms by a given fraction in a given proportion of individuals, equivalent to random treatment	Better modelling of transmission by age, immunity, worm mating. Stochastic model
Gurarie et al.		Deterministic	Kenya	Yes	Investigating MDA success in different scenarios using a modelling framework	Snail transmission compartments	None	A fraction of adult worms are killed by each treatment	Consideration of snail dynamics.
Soil-transmitted helminthiasis									
Coffeng et al. (hookworm only)		Stochastic,, individual	Vietnam	Yes	Developed WORMSIM, a new generalised framework for modelling transmission and control of helminths	Environmental reservoir	Gamma distributed total egg output, two scenarios: high or low variation in host susceptibility	Participation is either random, fully systematic or a mix.	Lifespan of eggs in the environment, MDA coverage over different age groups
Truscott et al.		Deterministic	India (*Ascaris*), St Lucia (*Trichuris*) and Zimbabwe (hookworm)	Yes (*Ascaris* only)	Fitting against multiple treatment rounds data	Pool of environmental infective material, exponential decay	Negative binomial distribution of worms in individuals	All individuals have a probability of receiving treatment	Understanding spatial and age heterogeneity, systematic non-compliance
Trachoma									
Gambhir et al.		Deterministic	Tanzania and Gambia	No	Including MDA interventions into the modelling framework	None	None	A subset of the infected group are moved to the susceptible compartment	Validating against multiple datasets, better modelling of immunity
Liu et al.		Stochastic compartmental	Niger	No	Constructing a stochastic transmission model including different ways of modelling each observation by fitting to TF only or to TF, TI and PCR	None	None	All individuals have a probability of receiving treatment	Further fitting to intervention data.

**Table 3:** Summary of modelling techniques used, IDM diseases

Paper	Model	Fitted to data from:	Predictions tested?	Technical advances	Model accounts for			Next steps
					Vector/environment dynamics	Heterogeneity in risk	Access to interventions	
Chagas disease								
Peterson et al.	Deterministic	Parameter values were set according to the literature	No	Formulating a transmission model and analysing the consequences of varying standard assumptions on the transmission cycle	Deterministic vector dynamics with animal hosts in some modelling scenarios	None	Not applicable - vector control only	Develop two independent transmission models. Estimation of changes in transmission rates
Human African trypanosomiasis, Gambian form								
Pandey et al.	Deterministic	Boffa, Guinea	Yes	Data cannot identify whether there is an animal reservoir. But in the presence of animal reservoir, there is high risk of re-emergence of HAT as public health problem.	Includes tsetse and animal compartments	None	All individuals have a probability of receiving treatment	Evaluating 2020 goal in other foci and impact of heterogeneity in human exposure to tsetse.
Rock et al.	Deterministic	Bandundu, DRC	No	Data supports the existence of an unscreened, high-risk population, but cannot identify whether there is an animal reservoir	Includes tsetse and animal compartments	High risk and low risk human compartments	Randomly participating and non-participating human compartments	Projecting impact of vector control in DRC
Leprosy								
Blok et al.	Stochastic individual	India, Brazil and Indonesia	Yes	Applied SIMCOLEP to predict future leprosy incidence in India, Brazil and Indonesia	Not applicable	Susceptibility: 20 % of population is susceptible; Type of leprosy: MB vs PB; Contact structure: general population vs within households	All individuals that have been diagnosed with leprosy receive MDT treatment. Probability of being diagnosed is determined by passive case detection delays and possible active case finding activities.	Assess which additional interventions are needed to meet the goals
Brook et al.	Statistical	604 analytic districts in India	No	Enhanced active case finding was associated with a higher case detection rate	Not applicable	Not applicable	Not applicable	Develop independent stochastic compartmental transmission model
Crump & Medley	Statistical	Thailand	Yes	Back-calculation can estimate the number of undiagnosed cases from diagnosed incidence rates	Not applicable	Not applicable	Not applicable	Consideration of gender and age. Analysis of other countries.
Visceral leishmaniasis in the Indian sub-continent								
Chapman et al.	Statistical	Bangladesh	No	Estimating durations of asymptomatic and symptomatic infection	Not applicable	Proportional hazards model for different risk factors including age, sex and bed net use	Not applicable	Developing a transmission model.
Le Rutte et al.	Deterministic	India and Nepal (KalaNet)	Yes	Developed three model structures, each with a different reservoir of infection, all fitting the data.	Vector population, deterministic.	Age-dependent sandfly exposure.	All individuals have a probability of receiving diagnosis, treatment, and vector control (IRS).	Implement best model structure in stochastic individual based model. Explore effect of additional interventions.
				Added heterogeneity in sandfly exposure.				
				Applied models to predict future VL incidence with current interventions.				

#### The importance of epidemiological settings

As expected, the details of an epidemiological setting, in terms of baseline prevalence, heterogeneities in risk by age and across the population and in terms of program implementation, are crucial in determining program success. The analyses of the PCT helminthiases in particular highlight that, in areas with different transmission rates, even with the same helminth (and vector), very different combinations of interventions are required to achieve control or elimination. As these models are developed further and linked more closely with programmatic activities, there are opportunities to better develop interventions aligned to local conditions.

The importance of epidemiological setting means that because these diseases are spatially heterogeneous, sampling for the impact of control is non-trivial, and low regional levels of infection may not be indicative of low transmission across an area (as illustrated by sub-national data for leprosy). A spatially heterogeneous transmission landscape (as is the case for NTDs) combined with some level of inevitable heterogeneity in how interventions are delivered and received is likely to lead to further heterogeneities in the levels of transmission following years of interventions. This may result in ‘hot-spots’ where additional interventions are required,. Although it may be difficult to identify or predict all hot spots, the modelling can demonstrate how the presence of hot spots contributes to heterogeneity and the need to adapt responses when such a location is detected.

#### Heterogeneities in risk and heterogeneities in access to care

A number of the analyses in this collection include models of both heterogeneities in risk of exposure and, importantly, access to care. Heterogeneities in transmission risk are more easily identified for helminth infections due to heterogeneities in pathogen load. For vector-borne infections there is also the possibility of measuring heterogeneities in exposure to insect bites. As demonstrated for helminth infections, two settings with similar prevalence but with very different levels of heterogeneity in risk may require quite different levels of interventions. In addition to these biological variations, particular behaviours can increase risk, whether it is children having higher exposure to STH, or adult males possibly having higher exposure to HAT. These will lead to differential impact of the available interventions.

These analyses have also highlighted that where high-risk groups are additionally less able to access care, or where there are other semi (or fully) systematic biases in access to interventions, this can have a large impact on the success of a programme. When the coverage rate is assumed to randomly reach any person with equal chance, the interpretation can conceal the fraction of a population that systematically misses the intervention. Models that include systematic factors in coverage are useful for relating to the practical realities of implementation, and thus help inspire operational improvements that reach the specific subpopulations previously at high risk for infection.

Modellers can characterise these heterogeneities in some settings, but of course not for all settings at all times. Given limited data, the modellers have been able to estimate some of the parameters that govern this variation in these settings, and have presented the sensitivity of their results to these underlying parameters.

#### Challenges of elimination as a public health problem versus “true” elimination

The first formal definitions of the public health targets for infectious disease were defined at a multi-disciplinary conference [67]. Since then the definitions have become somewhat corrupted: what is now commonly termed as “elimination” or “elimination as a public health problem” is more formally defined as control: “Reduction of disease incidence, prevalence, morbidity or mortality to a locally acceptable level as a result of deliberate efforts. Continued intervention measures are required to maintain the reduction”. The reason for the slippage in terminology is, as recognised at the conference, that political motivation to achieve elimination has to be developed and maintained. The current situation is potentially dangerous: most of the targeted NTDs are approaching “elimination”, but the models indicate that continued intervention is required to remain at the levels reached. The experience with leprosy indicates [68] that if achieving “elimination” results in a reduction in control efforts, at best progress is stalled and at worst disease will rebound. We need now to consider redefining the targets to be closer to true elimination: “Reduction to zero of the incidence of infection caused by a specified agent in a defined geographical area as a result of deliberate efforts. Continued measures to prevent re-establishment of transmission are required.” Modelling can help define these new targets.

### Next steps

#### Testing model predictions and model comparison

One of the strengths of this research project is the scientific robustness that comes from having independent modelling groups using different methods to address the same problems and the opportunities for testing predictions from multiple models. This has been most notably for HIV and malaria [69, 70] and there are lessons to be learned from the successes of these projects. For NTDs there has been some, limited, testing of model predictions against epidemiologic or programmatic data (Tables 2 and 3). This needs to be extended quite considerably in the next phase of this research project. By providing data from initial time points and asking the modellers to predict later time points, we will gain a better understanding of how the data informs parameter estimation and of particular weaknesses or strengths in the models. This will improve confidence in the model outputs.

Given the independent approaches within this research project and in the wider NTD modelling community, it is necessary to bring these results together and provide consensus information, whether through informal summaries (presented here), or through more rigorous methods. Possible approaches to arriving at consensus answers to the consortium’s research questions include:

1. analysis of the individual model projections, discussion on the differences and the possible causes of those differences and agreement on the most likely projection through discussion: *Model comparison*
2. arriving at a consensus model, through discussion on the strengths and weaknesses of each group’s approach for given geographical locales. This model will then be refitted to the baseline data and projected forward: *Consensus Model building*
3. mathematically combining the forecasts of each model through e.g., averaging. The cone of uncertainty for the forecasts is delineated by the upper and lower forecasts of each group. This is the approach of the international panel on climate change’s (IPCC) global surface temperature projections: *Ensemble Forecasting*

Each of these approaches has positives and negatives, which require further discussion. The joint onchocerciasis paper in this collection has brought together two modelling approaches which have been used for many years, and is gradually developing an understanding of what particular aspects of these models have generated different estimates of the number of rounds of MDA required to achieve particular targets [71]. This is a process of investigation, and through future model testing against multiple-timepoint programmatic data, a further quantitative assessment of the appropriate sets of assumptions and parameter sets can be made.

The development of a consensus model may be seen as a desirable aim from some stakeholders who would like a single answer to policy questions for very sound, practical reasons. However, built into this project is the recognition of the fact that different model assumptions and choices on how they are implemented can give different results and by using these different approaches we improve the scientific robustness of our conclusions. Indeed, arguably, for the diseases for which there has been very little previous modelling, independent analysis of the very few datasets which are available has led to a greater range of model assumptions than joint working would have generated, which builds more scientific robustness.

Ensemble forecasting, bringing together different models and weighting their output, is the current state-of-the-art in climate forecasting, and has been done to some extent in epidemiological modelling, but the weighting of the different models is challenging.

In the short term we hope to progress in our understanding of the different outputs of these models through carefully managed model comparison in order to provide consensus guidance on the key policy questions.

#### Data

As with all epidemiological modelling, there is a need for the models to be informed by high quality clinical and epidemiological data. The research and implementation community has been very supportive of this work so far, and there will be a greater number of re-analyses of old data, as well as analyses of new data, in the future. Part of our role is to improve access to these data for other modellers both now and in the future. We are currently collating a catalogue of the data that is used in our studies, and aim to facilitate access to these data for other modelling groups. It is important to remember that there are limited datasets currently available for modelling NTDs, and we should not be complacent that if we have modelled the few datasets available that we have a full understanding of the dynamics of these diseases. In particular, the models are very poor at replicating the behaviour of systems at low prevalence due to the high variability in potential outcomes. This will be a particular challenge for the future.

Model-informed data collection is a desirable outcome of this work, as it will broaden our understanding of the epidemiology [72, 73] and improve control. Some groups are actively seeking out such studies or are involved in the design of studies with these goals in mind, such as the Tumikia study in Kenya [74], which investigates the possibility of interrupting STH transmission though MDA. There are a number of similar activities across the nine NTDs.

The interpretation of raw data is sometimes hampered by issues with current diagnostic techniques. For example, models of helminth transmission are usually based around representations of worm numbers within hosts, but the connection between worm burdens and the output of egg-counting diagnostic techniques, such as Kato-Katz, or microfilarial counts are not well characterised, although it is known that sensitivities can be quite low. Newer diagnostics may provide more sensitive methods, but the quantification of load may be lost. It is therefore essential that the models are informed by the individual-level data on the relationship between different diagnostics, as well as tested against population-level intervention data using these diagnostics, not only to data using older methods. Any clinical or field trial of a diagnostic is an opportunity to work with the study designers to ensure that key variables are collected measuring model parameters linking the detection characteristics to immunology and with multiple diagnostic methods. The additional study data may come at no added cost or additional funds may be required for collaboration on a broadened scope. Timing is critical as many of the NTDs drop in incidence and research focus may shift elsewhere. At the same time data are more critical to providing a useful degree of certainty in the projections of low transmission levels.

For the IDM diseases, diagnostics are often poor at identifying active infection, and interpreting case data requires an understanding of the underlyling ‘effort’ in detecting cases. For these diseases it is important that analyses of such data are informed through close discussions with those who collected or collated the data. The quantification of underlying trends in incidence from case data requires a good understanding of the incubation period and the likely pathway from onset of illness to care, and how this varies by setting an by, for example, age, sex and socio-economic setting. It may be that this will never be quantifiable, and therefore independent measures of exposure, such as serological surveys, will be needed to assess program success and, importantly, evaluate local elimination.

#### Practical utility of models for research and public health community

For many of the papers we have released the code underlying the models. The remaining groups have also committed to releasing their code within the next months. The aim is to release the models in a format that expert epidemiological modellers can use now and in the future. This is to ensure that the work presented here is repeatable science, and that others can build on the work initiated here.

There is an admirable increasing trend for epidemiological model code to be realised and this generates some interesting points of discussion. Many of the models have been built for the analyses published in the collection and are subject to continuing development. They are already being altered to incorporate new intervention tools as they emerge such as the triple drug for lymphatic filariasis and oral stage-independent drugs for HAT, in order to simulate possible impact before they are rolled out.

Publishing the model code increases our collective responsibility to foster the acquisition of technical skills for anyone seeking to learn to use them [75]. The configuration of the models and the preparation of input data require knowledge of internal model structure and a large amount of statistical data processing if the model is to be adapted to any specific setting. Simply making educational resources known can efficiently guide new model users to the appropriate classes, lectures, literature, etc. We hope that the release of these models will stimulate opportunities for more collaborations and knowledge sharing, particularly with researchers in endemic countries. The value of the time invested in the formal and informal collaborations that will arise from them must be regarded as precious.

Of course, any model can be inadvertently misused giving misleading outputs and, as they have been released in its current form they need expert use. The original developers of the models currently lack the capacity for technical support ordinarily provided by a commercial software company, and the code should not be viewed as being produced for that level of use. The question still remains whether these models should be made available for local policy decision by development of more user-friendly interfaces, and also whether modelling expertise is required at that level [75]. For the moment, most of these models are not yet sufficiently validated to provide that local level of precise policy development, but through increased model testing and comparison that may become possible in future, provided they are sufficiently informed by, and tested against, the right data.

## Conclusion

This collection of research papers represents an important step forward for the evidence base for control and elimination of NTDs. They highlight settings where the 2020 goals, and even true elimination, are likely to be achieved using the current strategies. They also indicate that there are likely to be additional combinations of interventions required in other settings. These results do not provide the evidence for dramatic changes in policy, but can guide thinking and provide indications of ways forward which can be tested in future studies and analyses. The overarching messages of the models are highlight the importance of

- heterogeneity in risk of infection (and reinfection) and identifying which groups may maintain transmission as overall levels are reduced.
- heterogeneity in access to and acceptability of interventions, and possible systematic or semi-systematic patterns in any lack of coverage.
- considering transmission rates when considering strategies and endpoints
- clarity on the end goal of these programs and the development of strategies to maintain the gains achieved through elimination as a public health problem.

Through continuing collaboration across this team of modellers and their partners these researchers aim to provide further quantitative analyses which will assist the global effort to reduce the burden of NTDs towards the 2020 goals and beyond.

## Abbreviations

ALB: Albendazole
APOC: African programme for onchocerciasis control
DALY: Disability-adjusted life years
DEC: Diethylcarbamazine
DRC: Democratic Republic of Congo
GPELF: Global programme to eliminate lymphatic filariasis
HAT: Human African trypanosomiasis
IDM: Intensified disease management
IRS: Indoor residual spraying
ISC: Indian subcontinent
IVM: Ivermectin
KA: Kala-azar
LF: Lymphatic filariasis
LLIN: Long-lasting insecticidal nets
MDA: Mass drug administration
MDT: Multidrug therapy
NTDs: Neglected tropical diseases
OCP: Ochocerciasis control programme in West Africa
OEPA: Onchocerciasis elimination program for the Americas
PCT: Preventive chemotherapy diseases
PKDL: Post-kala-azar dermal leishmaniasis
PRET: Partnership for the rapid elimination of trachoma
SAC: School-aged children
SIS: Susceptible-infected-susceptible model
SWB: Stratified worm burden model
STH: Soil-transmitted helminths
TF: Trachomatous inflammation-follicular
TT: Trachomatous trichiasis
VC: Vector control
VL: Visceral leishmaniasis
WASH: Water, hygiene and sanitation
WCBA: Women of childbearing age
WHA: World health assembly
WHO: World Health Organization
